# Supervision of community health workers in Mozambique: a qualitative study of factors influencing motivation and programme implementation

**DOI:** 10.1186/s12960-015-0063-x

**Published:** 2015-09-01

**Authors:** Sozinho Daniel Ndima, Mohsin Sidat, Celso Give, Hermen Ormel, Maryse Catelijne Kok, Miriam Taegtmeyer

**Affiliations:** Department of Community Health, University Eduardo Mondlane, Maputo, Mozambique; Department of International Public Health, Liverpool School of Tropical Medicine, Liverpool, UK; Royal Tropical Institute, Amsterdam, Netherlands; Faculty of Medicine, University Eduardo Mondlane, Salvador Allende Avenue, 702, Maputo, Mozambique

**Keywords:** Supervision, Support, Motivation, Community health workers, Mozambique

## Abstract

**Background:**

Community health workers (CHWs) in Mozambique (known as Agentes Polivalentes Elementares (APEs)) are key actors in providing health services in rural communities. Supervision of CHWs has been shown to improve their work, although details of how it is implemented are scarce. In Mozambique, APE supervision structures and scope of work are clearly outlined in policy and rely on supervisors at the health facility of reference. The aim of this study was to understand how and which aspects of supervision impact on APE motivation and programme implementation.

**Methods:**

Qualitative research methodologies were used. Twenty-nine in-depth interviews were conducted to capture experiences and perceptions of purposefully selected participants. These included APEs, health facility supervisors, district APE supervisors and community leaders. Interviews were recorded, translated and transcribed, prior to the development of a thematic framework.

**Results:**

Supervision was structured as dictated by policy but in practice was irregular and infrequent, which participants identified as affecting APE’s motivation. When it did occur, supervision was felt to focus more on fault-finding than being supportive in nature and did not address all areas of APE’s work – factors that APEs identified as demotivating. Supervisors, in turn, felt unsupported and felt this negatively impacted performance. They had a high workload in health facilities, where they had multiple roles, including provision of health services, taking care of administrative issues and supervising APEs in communities. A lack of resources for supervision activities was identified, and supervisors felt caught up in administrative issues around APE allowances that they were unable to solve. Many supervisors were not trained in providing supportive supervision. Community governance and accountability mechanisms were only partially able to fill the gaps left by the supervision provided by the health system.

**Conclusion:**

The findings indicate the need for an improved supervision system to enhance support and motivation and ultimately performance of APEs. Our study found disconnections between the APE programme policy and its implementation, with gaps in skills, training and support of supervisors leading to sub-optimal supervision. Improved methods of supervision could be implemented including those that maximize the opportunities during face-to-face meetings and through community-monitoring mechanisms.

## Introduction

Numerous countries around the world have established community health programmes as a means to expanding access to health services among vulnerable populations, and these programmes are considered a vital component of reaching the health-related Millennium Development Goals [1]. With the shift towards the sustainable development goals and emphasis within these on equitable universal health coverage [2], there is an increasing need to understand how best to implement community health worker (CHW) programmes. CHWs, in Mozambique called Agentes Polivalentes Elementares (APEs), are an important component of health service provision in rural communities in Mozambique [3, 4]. There is a scarcity of literature regarding APEs in Mozambique, despite the APE programme having been established over three decades ago and having a clear impact on population health [5]. The initial APE programme (developed in 1978) faced challenges which resulted in the interruption of programme implementation in the mid-1990s. Primary concerns were that the APEs felt abandoned, due to almost non-existent supervision and a progressive decrease in support from the National Health Service, although many continued to receive drug and supply kits [6].

During this period, different APE-training curricula, implemented mainly by non-government organizations (NGOs) supporting the Ministry of Health (MoH), resulted in CHWs with wide variations in their scope of work mainly taking care of single “verticalized programmes” such as HIV/AIDS or tuberculosis [4]. NGOs implemented a system of subsidies and provided additional incentives for their own APEs, which led to frustration among volunteer APEs. Due to limited access to health services, community members started to demand more curative services from APEs which led some of them to become kind of “private health care providers”, charging small fees for their services. Both government-trained APEs and APEs and other community activists working on health-related issues that were trained and deployed by NGOs lacked supervision from the MoH. There was no appropriate monitoring of health activities at the community level nor were there any established indicators for evaluation purposes, constraints that ultimately led to the temporary suspension of the programme and the roll out in 2010 of a revitalized APEs programme by the MoH [4, 6].

In this revitalized model, APEs receive a 4-month residential training covering health promotion, disease prevention, testing and treating malaria in children and adults, diagnosing diarrhoea and dehydration, using oral rehydration solutions and diagnosing and treating acute respiratory infection in children. Additionally, APEs are trained to provide first aid and to detect danger signs in children, adults and pregnant women [4]. This training does not include explanation of the roles and expectations regarding supervision but does outline reporting requirements. APEs are volunteers who commit to certain terms through a “contract” which outlines their right to an allowance or subsidy and free health care at the local primary health centre or dispensary. While the subsidy is not linked to performance, in practice, it may delay or may be withheld if district or provincial reports are incomplete.

The APE programme has established protocols for programmatic supervision involving interaction between the province and district supervisors, district and health facility supervisors and health facility supervisors and APEs. Supervision of APEs is explicitly described as the responsibility of health workers, usually qualified nurses, from the health facilities of reference for a particular catchment area [4, 7]. Each supervisor looks after a group of APEs assigned to a particular health facility of reference (usually five to eight), and these APEs should ideally be working in an area between 8 km and 25 km from the health facility of their reference, close enough to allow APEs to visit health facilities monthly and face-to-face community visits for supervision and support from the health system staff to happen quarterly [7]. During planned health facility supervision of APEs, a checklist is used which covers several areas, including whether APEs have particular commodities available, have the tools they are expected to have and if they are completing and recording their duties correctly [8]. APEs are supposed to refer patients to their supervisors, bring monthly reports and collect their drugs and supply kits from the health facility of reference. The policy states that supervisors are in turn trained and supervised by APE programme managers from the district health directorate (district supervisors), who are also expected to provide technical support and visit APEs and their communities on a quarterly basis. While the facility staff have dual roles at the health facility, the district staff are APE-programme-specific. Finally, provincial supervisors exist under the umbrella of the national APE programme coordinator, and these may also visit districts and APEs [8].

Despite revitalization of the APE programme, considerable challenges remain regarding its successful implementation, including the supervision system. In available reports, a number of barriers were described about the human resource management of this cadre [3, 9]. Of particular note were the weak monitoring, supervision and feedback systems; the dual roles for APE supervisors as health facility workers; the allocation of resources for transportation/fuel; and weak referral systems.

Supervision has been linked to motivation and performance of health care workers. Motivation is defined as “an individual’s degree of willingness to exert and maintain an effort towards an organization’s goals” [10] and is a critical determinant of health worker performance [11]. Studies have shown that supervision can improve health worker performance, at least in the short term. In addition, supervision has been shown to be a mechanism facilitating professional development, improving health workers’ job satisfaction and increasing motivation [12]. International stakeholders, selected for interviews based on their range of programme and research experience in diverse settings and continents, also identified supervision as a key intervention to improve the retention and motivation of CHWs [13]. Supervision has been identified as a measure to enhance CHW motivation and performance in many settings [14, 15]. A recent review on supervision of CHWs found that regular supervision with supportive approaches, including community-monitoring, quality assurance and problem-solving, may be most effective in enhancing CHW performance [16]. However, data on CHW supervision is sparse, and there is a need to collect more data on the experiences of CHWs and their supervisors so as to better identify and understand the key components of supervision in practice and the links to enhanced CHW performance [16, 17]. Also, for the Mozambican context, the exact factors related to supervision contributing to motivation of APEs are not well explored yet. Therefore, the study set out to identify factors related to the organization and implementation of APE supervision and how these influence APE motivation and ultimately performance.

## Methods

Data collection was conducted as part of a multi-country study under the REACHOUT consortium (www.reachoutconsortium.org) which has the goal of improving the equity, effectiveness and efficiency of community health workers in six countries, including Mozambique. It was carried out in Manhiça and Moamba districts using qualitative approaches. Data collection was done from August to beginning of September 2013. The selection criteria of these districts were based on geographical situation, having an established APE programme and distances. A qualitative study was chosen as most appropriate to reach the objective of this study, because it could obtain in-depth insight into how supervision of APEs was conducted and what made it facilitate or hinder APE motivation and performance [18, 19]. The study involved four types of purposefully sampled respondents: APEs (18 out of a possible 41 APEs were selected across the two districts), health facility supervisors (5 out of a potential 14 across the two districts), district supervisors (both district-level supervisors were included) and community leaders (6, each representing a locality with several communities or villages and coming from the communities that the APEs and supervisors served). Respondents were selected based on geographical location, distance to health facilities and to ensure diversity in gender, age and job experience and were identified with the help of district-level and health facility staff.

The study was conducted by a trained and experienced research team through in-depth interviews (IDIs) focusing on aspects related to supervision, motivation and performance. We used IDIs with the purpose of exploring strengths and weaknesses of community services and barriers and facilitators to APE performance. The IDIs assured that participants could be interviewed in their rural communities and avoided issues of hierarchy affecting group discussion [20].

Semi-structured topic guides were developed in English first (as part of the multi-country study), translated into Portuguese and back-translated for consistency. IDIs with APEs included questions on recruitment, incentives, motivation, training and support, roles and tasks, supervision and communication. For supervisors, we added questions on barriers and enablers of supervision and on management and training issues. Community leader interviews included issues of community engagement and ownership, selection and support of APEs, knowledge of APEs’ roles and expectations. The topic guides were piloted in an area that was not included in the study and adaptations to probes and questions were made. For some APEs and community leaders (those with no or only primary education), it was necessary for interviewers to explain concepts in the local languages of Ronga and Xichangana, despite Portuguese fluency being a selection criteria for APE recruitment.

Standard procedures and tools were used to ensure correct and complete data. Daily debriefing sessions with all data collectors were held to discuss key findings, refine lines of inquiry and summarize extensive field notes and observations. The interviews were digitally recorded, and Portuguese transcripts were made of each IDI by the interviewers who also rechecked each other’s transcripts against the recordings for quality assurance. The transcripts were independently read in Portuguese in pairs by a group of four researchers (SN, CG, HO, MS) to identify key themes and develop a coding framework. This process used open-coding [21], combined with a pre-defined framework of factors that could influence CHW performance [15]. A sample of transcripts was further translated into English to allow anglophone authors to input and review the coding framework. Transcripts were coded in Portuguese using NVivo (v.10) software, and emerging themes were discussed and the coding refined based on team consensus. The coded transcripts were further analysed, “charted” and summarized in narratives for each theme and sub-theme in Portuguese. A full report of the complete context analysis with extensive quotes, of which this study forms a part, was produced and translated into English [22], and additional queries for the purposes of this paper were run and narratives written directly in English. Study findings were validated with the two district health offices through informal feedback meetings and sharing and discussing a summary of the report in Portuguese. This study received ethical approval from the Institutional Review Joint-Board of the Faculty of Medicine of the University Eduardo Mondlane and Maputo Central Hospital (CIBS FM&HCM/07/2013) and from the Royal Tropical Institute (KIT), in Amsterdam.

## Results

We interviewed a range of APEs, health facility and district supervisors and community leaders. These were purposively selected from the two districts to represent different profiles, as outlined in our “Methods” section (for details, see Table 1). Overall, there were more males interviewed in each participant type, and the APEs were younger and more likely to be single than the supervisors or community leaders, with close to half of APEs having secondary education. In order to ensure confidentiality, we have not added potential identifiers such as gender, district or level of supervisor to the quotations from the small group of supervisors.

**Table 1:** Participant districts and profiles

		APE	Health facility supervisors	District supervisors	Community leaders
Total sample		*n* = 18	*n* = 3	*n* = 2	*n* = 6
District					
	Moamba	10	2	1	2
	Manhiça	8	1	1	4
Total		18	3	2	6
Characteristics					
Gender	Male	10	2	2	5
	Female	8	1	0	1
Total		18		2	6
Age	18–25	9	1	0	0
	26–35	3	1	2	0
	35–44	5	0	0	0
	>45	1	1	0	6
Total		18	3	2	6
Marital status	Married	6	3	2	6
	Single	11	0	0	0
	Divorced	1	0	0	0
Total		18	3	2	6
Education	None	0	0	0	2
	Primary	10	1	0	3
	Secondary	8	2	2	1
Total		18	3	2	6

From the data analysis, there were five key emerging themes related to the interaction between APEs and supervisors that illustrate a range of factors that participants felt influenced the motivation and performance or both groups. First, supervisors acted as important links between health systems and communities; second, there were gaps in policy content and a mismatch between policy and practice leading to irregular and infrequent supervision; third, the skills and approach to supervision were important in determining APE motivation; fourth, supervisors themselves felt largely unsupported; and lastly, we also found that APEs received support and monitoring from the community and a high level of community engagement in “supervising” their work.

### Supervisors are links between health systems and communities

Supervisors provide an important link between the APE programme and the health system on the one hand and between the programme and communities on the other. In both districts, the supervisory system appeared to be organized into the four levels described by policy: APE, supervisor at health facility of reference, district supervisor and provincial supervisors with a largely functional reporting system at district and provincial levels. However, the collecting of data for monitoring purposes which is transferred in an upwards direction, was often not discussed with, or fed back to, APEs who felt unaware of their own performance and the state of the programme in general. Supervision was instead mentioned as a data-gathering exercise for monitoring and evaluation of the APE programme.

“They tend to evaluate using the monthly summaries. Observe the number of cases treated, number of pregnant women, new-borns, adults and children, transferred cases, people who had been treated for malaria, number of lectures and participants. They want to know how many people I have attended to, and if this is to reduce or increase.” (APE, 23 years old, female)

While general programme data were not fed back, this APE also described requesting and receiving immediate feedback:

“I think that they evaluate me through supervision. When they come here they want to know what will work, see the log book, seeking to know if I have doubts. Soon when coming to supervision I have to express my doubts; the supervision facilitates me, and I know how my work is going.” (APE, 28 years old, male)

Both supervisors and APEs agreed on the potential of supervision for capacity strengthening of the APEs. Supervisors described the need to teach and mentor APEs and often helped them with their monthly reports, technical advice on promotion or prevention and with clinical queries. The support and ongoing education provided was appreciated by the APEs as expressed by these two representative quotes:

“When I receive supervision visits, I ask about things that I cannot do, and they show me how to do them. So we learn things we don’t know how to do during the supervision, and we like that.” (APE, 26 years old, male)

“Supervision is always seen as good and important for the type of work we do. We APEs are not ‘doctors’. We have very limited knowledge in many things we do and a supervisor must always be there to teach new things and remind us about what we have learnt.” (APE, 28 years old, male)

The presence of supervisors in the community was welcomed and was seen by APEs and community leaders as enhancing the credibility of the programme as well as that of APEs in their respective communities. Conversely, their absence undermined perceptions of APEs and of the health system as pointed out by this APE:

“Even my community will not respect me when they don’t see my superiors coming here.” (APE, 23 years old, female).

Additionally, when subsidies were not paid or stock-out of medicines occurred, the supervisors, as the accessible human face of the system, had to provide explanations and negotiate. The supervisors felt caught between administrative and management issues around APEs’ subsidies that they were not in a position to solve and perceived this as undermining their credibility to respond on behalf of the health system they represented:

“In this one year only 22 APEs worked, and three people already gave up. I’m sure that if the process continues so many will give up, only because of the subsidy. The first complaint is that it is little, and even then it does not appear monthly; the delay influences them a lot. …Every day that passes there is a message that comes to us asking about the subsidy. They always send messages asking about the allowance: when will it come out? We are now on 21 August, and this is the fifth month that we do not have a subsidy.” (Supervisor, 28 years old)

Delayed subsidies limited the motivation of supervisors to visit and oversee APEs, as supervisors did not feel comfortable demanding more work knowing that APEs were not receiving their subsidies on time and were demotivated.

“I as supervisor I do not feel good when I go to a community for supervision and require a lot from the APE while after that the APE will ask me about the subsidy and the answer will always be the same: we are still dealing with that but they will never receive such money.” (Supervisor, 28 years old)

### Supervision is irregular and infrequent

According to APE programme guidelines, the frequency of supervision for district to facility level is quarterly and for facility level to APE level is monthly in two ways: during report delivery and collecting of drug kits at the health facility and during actual supervision visits at the community level. Although the supervision system is organized according to a timetable, both APEs and supervisors pointed out that it rarely occurred on the scheduled dates and was both infrequent and irregular. The lack of transportation, long distances to travel and difficult access to communities were identified as factors that negatively affected regular supervision. In some cases, supervisors spent more than 2 months without carrying out supervisory activities. While most supervisors identified lack of resources as the problem, some also pointed to poor coordination within district health directorates as the cause of delays in the allocation of fuel and other logistical needs for regular supervision to take place. Other issues mentioned included pending maintenance of their motorbikes as explained by this supervisor:

“We also have difficulties with transportation in that a motorcycle sometimes does not have fuel. I waited one month following the request without receiving the fuel. Also there is the issue of faults: most of the time or almost always I have to do maintenance of the motorcycle, because waiting for the district means stopping, and how many activities will that affect? Rather than waiting for the district, I’m going after it myself.” (Supervisor, 26 years old)

The same supervisor describes the impact of dual roles, a sentiment echoed by all the other supervisors interviewed:

“Working as an APE supervisor has not been easy, because I’m alone, and when I go out for supervisory activities, other activities are stopped, but I cannot let the activities of the APEs suffer because of the others. I always have to run behind time. Sometimes I cannot do oversight on the scheduled dates, and I have to go after working hours.” (Supervisor, 26 years old)

Roughly 2 years since the beginning of the programme, some communities and APEs had only received two supervisory visits. The infrequency and irregularity of supervision was felt by many participants to negatively affect the performance of the programme and of individual APEs in achieving objectives. This situation can make APEs demotivated, reduce the communication between APEs and health facility coordinators and reduce the effectiveness and efficiency of the programme interventions, as this APE pointed out:

“I had my last supervision in June last year and so far not yet had any other visit that demotivates me because it seems that I was forgotten.” (APE, 23 years old, female)

### Supervision skills and approach are important determinants of motivation

Supportive supervision emerged as a strong determinant of motivation among the APEs, and an approach that relied on fault-finding rather than support was reported as a strong demotivating factor. While APEs identified this as a problem, the supervisors did not. Many appeared to regard checklists as a key (and only) tool required for supervision, determining fault-finding as the correct way of supervising and feeling comfortable with it. This approach, however, may have made it harder for them to identify and deal with problems at all. Some APEs stated that when a supervisor is mainly looking for the bad things, they tended to hide their gaps during subsequent supervision visits.

“When he comes here he looks at the record book and then just focuses on what I did wrong. He says that he is doing that to support me but I don’t think so. Sometimes I prefer to not share my gaps with him or even if he couldn’t come here”. (APE, 26 years old, male)

In fact, most of the supervision described by the APEs was based on checklists with the supervisor focusing on issues present on the list (such as number treated, number of home visits, number of referrals and reporting), and roles that were not on the list (such as health promotion meetings or topics) were described as not being taken into consideration; neither was constructive feedback given, as illustrated by this typical quote from an APE:

“My supervisor has supported me in many ways and I believe he has good skills for his job, but when he comes to supervise me he only takes the log book and begins to observe and often does not give me report about my work … where I should improve” (APE, 45 years old, female)

While few APEs had experience or expectations of supportive supervisions, they identified factors and skills that motivated them and that are traditionally linked to this approach, such as listening, praising good work, joint problem-solving and non-judgemental attitudes.

### Supervisors lack training for the role and many feel unsupported

Despite the fact that APE supervisors should be trained according to the manual, it was found that training of health facility supervisors was overly focused on how to complete checklists. In addition, supervisors mentioned that individuals who had been trained had been transferred from one health facility to another or from one district to the other, leaving the supervision tasks with staff unaware of the roles and tasks of the APE programme as this supervisor explained:

“I am supervisor since the APE programme was revitalized here, but I have never been trained. They (superior level) gave me some tips during my supervision on how to conduct a good APE supervision, but like others I may one day be transferred to another site and be replaced by someone else who doesn’t have any skills or even knowledge regarding the programme”. (Supervisor, 34 years old)

Districts supervisors may be in a better position in terms of supervision skills than health facility supervisors, as they are full-time APE programme managers at the district level. Despite this, they did not receive any initial training for supervision but learnt by doing.

“Most of the things I know I was not taught, I acquired supervision skills by doing what I was supposed to do and by reading all the tools regarding APEs supervision”. (Supervisor, 32 years old)

Some supervisors learnt how to conduct supervision through their own supervision from their superior level, and this they also described as focused on fault-finding. Thus, the APE supervisors used the same approach when supervising the APEs. Health facility supervisors were supported by district supervisors providing logistical arrangements and occasionally accompanying visits to the field, often taking the opportunity offered by researchers, NGOs or other visitors travelling to see APEs. There were no arrangements described for refresher training of supervisors, meetings of supervisors or other sources of support and capacity development.

### Community-monitoring

The communities were also involved in monitoring and accountability of APEs, and the health facility supervisors and the district supervisors linked with community leaders to find out how the APE is working. Regarding this, a supervisor said:

“We coordinate with the community leaders. For example, the community leader controls the activities, so I have to be informed about how they are working. He gives me the information. They sometimes go with the APEs during the lectures about health promotion and disease prevention to see how the APE is working with the community.” (Supervisor, 28 years old)

This was backed up by interviews with community leaders, who described the community as having a governance function that provided oversight and a degree of control, as well as support, of the APEs’ work, ensuring that drug kits were opened in their presence, that feedback was gained from wider community members and that reports were reviewed before submission.

“In the meetings we used to have here in the community we ask the population if our APE is working well or not and we give the report to the nurse when she comes.” (Community leader, 78 year old)

“We are always available to help our APE and we have an obligation to see how it is working. For example, the drugs kit can only be opened in our presence to ensure control of the drugs because they must be used for this community and for the goodness of this community and if we see something wrong we must report the health facility.” (Community Leader, 54 years old)

This APE also confirmed a high level of community engagement with monitoring his work:

“The community leaders and people here in the community follow all my work. They almost know everything that happens here in the community. Even my monthly reports that I send to the health facility they want to see first.” (APE, 26 years old, male)

Community-monitoring, while not formal health system supervision, represents a different kind of influence on APE motivation. In our context, it seems to be functioning alongside, and in some respects better than, formal supervision. Through its supportive approach, positive feedback and lack of fault-finding and checklists, it becomes more in line with how supervision might function to motivate community health workers and opens the possibility of a partnership that links this to formal supervision.

## Discussion

Our findings indicate that supervision was structured as dictated by policy documents, and APEs considered supervision and the accompanying mentorship as a key factor in maintaining their motivation. Supervision also provided APEs with a sense of belonging to a health system emphasizing the connection between them and their health facilities and an enhanced credibility in their communities. However, in practice, the supervision of APEs through community visits by supervisors was infrequent and irregular. When it did occur, supervision was perceived to focus more on fault-finding than being supportive in nature and did not address all areas of APEs’ scope of work – factors that APEs identified as demotivating. Supervisors from reference health facilities in turn also felt unsupported and perceived this to negatively impact on their performance as supervisors. They had a high workload in health facilities, where they had multiple roles, and they felt unable to dedicate appropriate attention and time to the APEs under their supervision. Lack of logistical and financial resources for supervision activities was identified as a challenge, and supervisors felt caught up in administrative issues around APE allowances that they were unable to solve, as these depended on higher hierarchical levels of the health system. Many supervisors were not trained in supportive supervision. Community governance and accountability mechanisms represent a potential for additional support but were only partially able to fill the existing gaps in supervision of APEs by health system staff.

The role that supervisors play in acting as a bridge between CHW programmes and the health system on the one hand and between the programmes and communities on the other is described elsewhere [17, 23, 24]. Supervisors in our study were aware that they needed to act as mentors and trainers. They were also aware of the responsibility of giving feedback on referrals and completing the monitoring and evaluation feedback loop. As reported in a study on community-based HIV care in Mozambique, when care becomes more technical, the role of supervisors as a bridge to the health system, able to explain new developments and tasks in a way that APEs with a lower level of education find accessible, becomes increasingly important [25].

The importance of a coordinated approach to supervision from the health system perspective is key if universal health coverage is to be achieved in an equitable manner and with services of sufficient quality [26]. However, supervisors felt that coordination was often lacking at the district level. Resource limitations and transport issues in rural settings have been raised as challenges by supervisors in rural South Africa, and altering the nature rather than the frequency of supervision may be one way of dealing with this challenge [16, 17].

Our findings strongly endorse the desire for supportive supervision approaches both from APEs and from supervisors, who feel under supported and lack role modelling of supportive supervision approaches themselves. Supportive supervision is a process described as promoting quality in health system functioning by strengthening relationships, focusing on the identification and resolution of problems and helping to optimize the allocation of resources – promoting high standards, teamwork and better two-way communication [27]. Such approaches are linked to improved health worker performance, motivation and increased and sustained job satisfaction [12], and this is borne out in a multiple case study of supervision at peripheral health posts in Guatemala that highlighted the importance of a supportive holistic approach as opposed to the prevailing model of managerial control [28]. The same holds true for supervision of community health workers. A recent review assessing the impact of CHW supervision found that of the available supervision strategies for CHWs, the ones that focused on a supportive, problem-solving approach were more effective. In addition, improving supervision quality had more impact than increasing the frequency of supervisory visits to the community [16]. Supportive supervision approaches have been shown to have a positive impact on malaria prevention and treatment by CHWs in India [29]. Conversely, a study of CHW motivation in Tanzania found that supervision that was perceived by CHWs as a sign of poor performance was a demotivator [30].

The proximity of communities and the relative remoteness of the health system and its representatives mean that communities often take on a governance and accountability role in relation to their local community health programme. Designing a supervision system based on approaches used within the formal health system may be inadequate unless it is adapted to build on links with the community as well [16]. Community-monitoring or supervision are likely to be important approaches to improve APE performance, given that they work in communities, are selected by them and feel responsive and responsible to them. The importance of listening to community and APE voices was highlighted by ethnographic research with APEs in Mozambique [31], while CHWs in Tanzania reported that families and communities supplement other sources of motivation by providing support [30]. The inSCALE study with CHWs in Uganda and Mozambique is currently conducting a three-arm trial comparing technology and community-supported supervision approaches (using village health clubs) with control groups that will provide more information on approaches to community supervision [32].

The importance of supervisor training skills and knowledge emerged as a key theme in our findings, and this has also been described in a review of supervision and mentoring for remote rural health workers [26]. However, the exact nature and content of training varies widely across contexts and often focuses on developing technical skills rather than examining values and attitudes, ability to understand and support individuals or group dynamics. The South African study based in an infant-feeding programme highlighted the importance of broader people management skills among supervisors and the need for additional training and support to supervisors that help them to perform these roles, enabling them to move away from checklists and endorsing a more supportive approach [17]. Training on supportive supervision should take into account that supervisors and supervisees are not blank in supervision knowledge and experience and should integrate and build on their current knowledge and skills. An ongoing study with lady health workers in Pakistan is investigating the training needs of supervisors after an evaluation revealed a lack of skills in supportive supervision. This study will assess impact on CHW performance in community case management of diarrhoea and pneumonia [33].

Our study has a number of limitations. We used qualitative methods and are thus unable to comment on the representative nature of our sample. The number of supervisors included in our study was small, and we are not able to present disaggregated data between health facility and district-level supervisors for reasons of confidentiality. The impact of CHW profile on issues of empowerment, ability to speak out to supervisors and understanding concepts of supportive supervision would require a larger data set and better balance of genders, with more targeted questioning to enable a full gender analysis to be conducted. APEs and community leaders for interviews were selected with the help of supervisors, although all available APEs in the districts were sampled, wherever possible.

### Recommendations

Our study suggests the need for developing a supportive approach to supervision as an important next step in the two districts. There is a need for solutions that are sustainable and viable for implementation in resource-constrained settings such as the study sites (which reflect the majority of districts in the country). A group approach to APE supervision combined with support and training for supervisors would ensure a supportive supervision strategy that efficiently uses the short time that supervisors have to conduct good supervision. This would also allow APEs to develop problem-solving skills as a group through peer support and supervisors to come together for a common training to explore and build on their existing strengths. Training APEs on what to expect from supervision, empowering them to seek advice and encouraging them to seek support from community-monitoring systems can further empower APEs as community agents.

## Conclusion

This study adds to the limited body of literature on supervision of APEs in Mozambique and discusses these in the light of data and evidence from other community health worker programmes. We identified the importance of regular supportive supervision as a key determinant to APE motivation and a potential way of improving APE and programme performance. A range of barriers to the supervision system under the revitalized programme should be addressed. APEs in the previous programme felt abandoned due to the lack of supervision, and this appears to be a recurring constraint in the revitalized programme. While some APEs indicated that they want more supervision, constraints in supervision frequency arose at both the facility and district levels, relating to budget and access issues but also to supervisors’ dual roles and limited means of transport. Using community-monitoring in motivating and empowering APEs represents a potential additional supportive mechanism but cannot address all of the identified issues without linkage to the formal health system and supervision structures as well as efforts to create supervision structures that are supportive and problem-solving rather than managerial and fault-finding. Tailored solutions would be required, as each district/community has its specific characteristics, and challenges are not always similar in all parts of the country. Furthermore, when supervision does take place, feedback and thus learning opportunities should be the focus so that both supervisor and APE can positively contribute to enhanced community health.
